# Gender Effects on Plasma and Brain Copper

**DOI:** 10.4061/2011/150916

**Published:** 2011-10-19

**Authors:** Joseph F. Quinn, Christopher Harris, Jeffrey A. Kaye, Babett Lind, Raina Carter, Thimmappa Anekonda, Martina Ralle

**Affiliations:** Department of Neurology, Portland VA Medical Center and Oregon Health & Science University, Portland, OR 97239, USA

## Abstract

The effect of gender on systemic and brain levels of copper is relatively understudied. We examined gender effects in mice and human subjects. We observed a trend to higher serum copper levels in female compared to male LaFerla “triple transgenic” (1399 ± 233 versus 804 ± 436 ng/mL, *P* = 0.06) mice, and significantly higher brain copper levels in female- versus male wild-type mice (5.2 ± 0.2 versus 4.18 ± 0.3 ng/mg wet wt, *P* = 0.03). Plasma copper was significantly correlated with brain copper in mice (R2 = 0.218; *P* = 0.038). Among human subjects with AD, both plasma copper (1284 ± 118 versus 853 ± 81 ng/mL, *P* = 0.005) and cerebrospinal fluid copper (12.8 ± 1 versus 10.4 ± 0.7 ng/mL, *P* = 0.01) were elevated in women compared to men. Among healthy control subjects, plasma copper (1008 ± 51 versus 836 ± 41 ng/mL; *P* = 0.01) was higher in women than in men, but there was no difference in cerebrospinal fluid copper. We conclude that gender differences in copper status may influence copper-mediated pathological events in the brain.

## 1. Introduction

Copper has been implicated in the pathological aggregation and neurotoxicity of beta amyloid (A*β*) [1] in Alzheimer's disease (AD). The evidence for “excess” circulating copper in AD has been reviewed recently [2] and copper-modulating therapies for AD are being evaluated [3, 4]. Although gender may influence the appearance of AD pathology, the effect of gender on copper status is relatively understudied. Some published reports have described gender differences in serum copper levels [5–7], but the effect of gender upon brain copper, which is more relevant to AD pathogenesis, has not been previously described. 

We tested the hypothesis that gender modifies both circulating and brain copper levels, with potential consequences for AD pathology, using samples from an animal model of AD and from human subjects with and without AD.

## 2. Materials and Methods

### 2.1. Transgenic Mouse Studies

Breeding pairs of wild-type and “triple transgenic” (3xTg) mice [8] were generously provided by Dr. Frank LaFerla, and offspring were raised in the Portland VA Medical Center Veterinary Medical Unit. Mice were maintained on AIN93 diet from the time of weaning, with ad lib deionized water, so dietary intake of copper was closely regulated. For these experiments, 7 female wild type, 7 female transgenic, 4 male wild-type, and 3 male transgenic mice were used. At the age of 14 months, mice were euthanized with terminal collection of plasma and with rapid harvest of brain tissue. Copper levels were determined in brain (bilateral frontal cortex) and plasma by atomic absorption spectroscopy. All procedures were approved by the Portland VA Medical Center Institutional Animal Care and Use Committee.

### 2.2. Human Subject Studies

Individuals with AD as well as healthy control subjects were characterized by clinicians at the Oregon Health and Science University NIA-funded Alzheimer's center. AD was diagnosed according to NINDS-ADRDA criteria [9]. Healthy control subjects were tested with neuropsychologic battery and with interview of a collateral historian to ensure that they are genuinely healthy controls. 

With appropriate Institutional Review Board approvals, subjects donated plasma by venipuncture and cerebrospinal fluid by lumber puncture. All lumbar punctures were performed in the AM fasting condition, in the lateral decubitus position. Both plasma and cerebrospinal fluid were frozen at −70 until copper measurements were performed by ICPMS.

However, plasma samples from AD patients and controls were collected in different tubes, with AD samples in heparin tubes and control samples in EDTA tubes (because the samples were initially collected under different protocols before being deposited in a repository). The use of EDTA (rather then heparin salt) collection tubes has been identified as a major source of variability in cross-sectional studies comparing blood levels across patient populations [2]. The analysis below consequently does not compare plasma levels across diagnostic groups.

### 2.3. Statistical Analysis

Group means were compared by two-tailed *t*-test or ANOVA, depending on the number of means being compared.

## 3. Results

**(1) Serum Copper in Mice**

**Figure 1:**
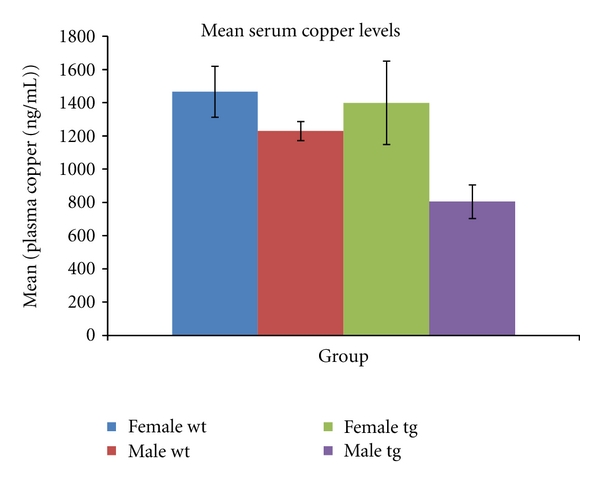
Serum copper levels (mean ± SEM) in triple transgenic (tg) and wild-type (wt) mice. Trends to higher serum copper in females are evident in both strains (*P* = 0.06 for female tg versus male tg; *P* = 0.19 for female wt versus male wt. *n* = 7 female wt, 4 male wt, 7 female tg, 3 male tg).

Serum copper is increased (*P* = 0.06) in female (1399 ± 233 ng/mL) compared to male (804 ± 436 ng/mL) 3xTg mice, and a trend in the same direction is seen in wild-type mice (1467 ± 128 ng/mL in females and 1230 ± 169 ng/mL in males, *P* = 0.19) (see Figure 1).

**(2) Brain Copper in Mice**

**Figure 2:**
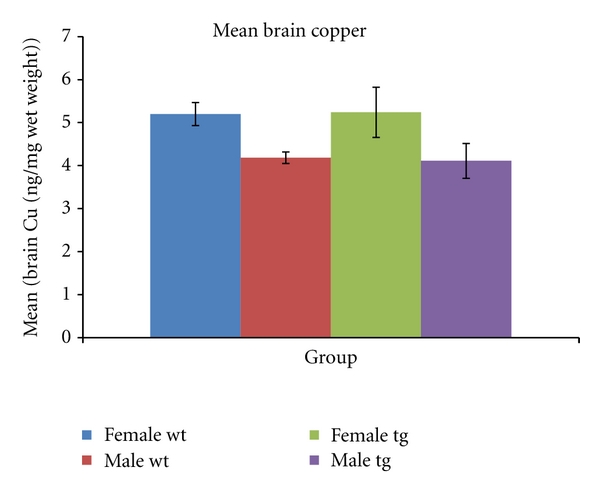
Brain copper levels (mean ± SEM) in triple transgenic (tg) and wild-type (wt) mice. Brain copper is significantly higher in female wt compared to male wt mice (*P* = 0.03), and a trend to higher brain copper in female tg compared to male tg mice is also evident (*P* = 0.15).

Brain copper is significantly increased (*P* = 0.03) in female (5.2 ± 0.2 ng/mg wet wt) compared to male (4.18 ± 0.3, *P* = 0.03) wild-type mice, and a trend in the same direction is seen in 3xTg mice (5.2 ± 0.5 versus 4.2 ± 0.8, *P* = 0.15; see Figure 2). Plasma copper was significantly correlated with brain copper in mice (R2 = 0.218; *P* = 0.038).

**(3) Human Subject Characteristics**

Control subjects were characterized as “young” (age 20–40, *n* = 11), middle-aged (age 41–60, *n* = 16), and old (age ≥ 60, *n* = 15). The old controls did not differ from the AD subjects in mean age (see Table 1) or in percentage of women. Subjects with AD had mild deficits, illustrated by mean MMSE = 17 ± 0.7 (see Table 1).

**(4) Plasma Copper in Human Subjects**

Plasma copper was not correlated with age among control subjects or AD patients. Plasma and cerebrospinal fluid copper were not correlated in any group.

**Figure 3:**
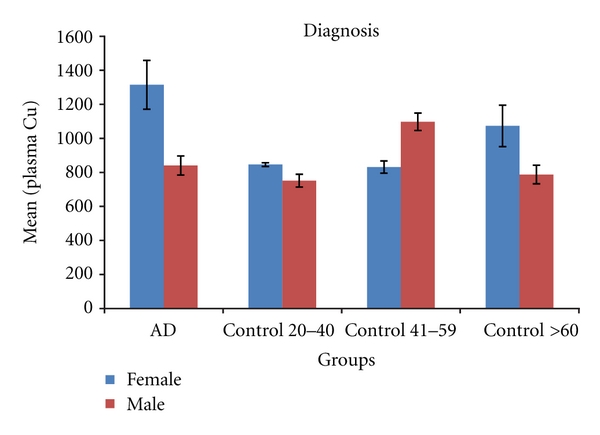
Plasma copper levels (mean ± SEM) in human AD and control subjects. Plasma copper is significantly increased in female AD (*n* = 11) compared to male AD (*n* = 23) (*P* = 0.005). There is a trend to increased plasma copper in old female control subjects (*n* = 6) compared to old male control subjects (*n* = 8) (*P* = 0.09) and in female middle-aged control subjects (*n* = 8) compared to middle-aged male control subjects (*n* = 8) (*P* = 0.13). When all healthy control subjects in each gender are combined, there is a significant difference in plasma copper (*P* = 0.01, see text).

Plasma copper is increased in human female (1008 ± 51 ng/mL) compared to male (836 ± 41) control subjects (*P* = 0.01) when all age groups were combined. When the control subjects were divided by age group, trends to higher plasma copper in females were appreciated in the middle-aged and old control subjects, but not the young subjects (Figure 3).

Among subjects with AD, plasma copper was significantly increased in female compared to male AD subjects (1284 ± 118 versus 853 ± 81 ng/mL, *P* = 0.005).

**(5) Cerebrospinal Fluid Copper in Human Subjects**

**Figure 4:**
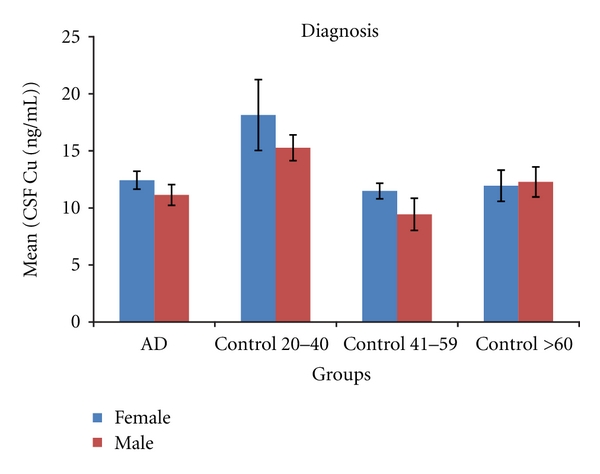
Cerebrospinal fluid (CSF) copper levels (mean ± SEM) in human AD and control subjects. CSF copper is significantly increased in female AD (*n* = 16) compared to male AD (*n* = 25) subjects (*P* = 0.04), but there are no significant differences in CSF copper between male and female healthy control subjects.

CSF copper is increased in human female compared to male AD subjects (12.8 ± 1 versus 10.4 ± 0.7 ng/mL, *P* = 0.01). No gender-specific difference in CSF copper was seen in control subjects considered as a single group (12.2 ± 0.7 versus 11.6 ± 0.52; *P* = 0.47) or when considered by age group (see Figure 4). There was also no significant difference between AD patients and control groups in CSF copper, consistent with a recent meta-analysis on this topic [2].

## 4. Discussion

These findings in both mice and human subjects are consistent with a small number of publications in human populations which have found an effect of gender on serum copper, with higher copper levels in women than in men [5–7]. The possible confounding effects of dietary intake of copper [10] or the use of copper-containing supplements [2] has been emphasized in other studies of human subjects. However, the demonstration of gender effects in mice, with strict controls on copper intake which are not possible in human studies, strengthens the argument that these gender differences are due to gender-specific differences in copper trafficking rather than differences in dietary intake.

In some transgenic mice engineered to express AD pathology, female mice are more prone to AD pathology than male mice [11], and most studies examining the effect of gender on AD risk in human subjects [12–14] find an increased risk in women (although there is some controversy surrounding this point, with one study finding increased risk in women only after age 90 [15]). In light of these observations, it is interesting to speculate that gender differences in copper status, perhaps related in some way to iron-regulatory mechanisms related to menstruation, might modulate these apparent gender effects on AD pathology. Beyond speculation, gender effects on copper may also be important considerations in the design, conduct, and analysis of clinical trials of copper-modulating therapy for AD. 

Further investigation of the consequences of gender-specific differences in copper status may be facilitated by clarification of the relationship between systemic and central nervous system copper. The significant positive correlation between serum and brain copper in the mice in this study supports the hypothesis that blood levels are relevant to brain levels of copper, and the concordance between serum and brain tissue results (with copper levels higher in females in both cases) provides further support for this view, at least with respect to frontal cortex. 

However, since brain levels are not an option in living human subjects, these experiments used cerebrospinal fluid as a surrogate for brain tissue. In the case of subjects with AD, the effects of gender on plasma and cerebrospinal fluid copper were concordant, suggesting a blood-brain relationship similar to that seen in the mice. However, the absence of a correlation between human plasma and cerebrospinal fluid copper in both AD and control subjects suggests that either plasma copper does not reflect brain tissue or that cerebrospinal fluid is not an adequate surrogate for brain tissue in this instance. Alternatively, it may be necessary to measure the proportion of CSF copper not explained by ceruloplasmin in order to appreciate plasma: CSF correlations, as reported by others [16].

## 5. Conclusions

These data add to existing evidence that female gender has an effect on blood levels of copper and provide new evidence that female gender may also have an effect on brain levels of copper. Gender effects on copper status may need to be considered in interpreting experiments, including clinical trials, which test the hypothesis that copper plays a role in AD pathogenesis and progression.

## Figures and Tables

**Table 1:** Human subject characteristics.

	Young control	Middle-aged control	Old control	AD
*n*	11	16	15	38
Age in years (mean ± SEM)	30 ± 2	50 ± 1.8	74 ± 1.8	70 ± 1.2
Gender (% female)	19%	50%	47%	34%
MMSE	30 ± 1.3	30 ± 1.2	29 ± 1.2	17 ± 0.7
